# Isoamericanoic Acid B from *Acer tegmentosum* as a Potential Phytoestrogen

**DOI:** 10.3390/nu10121915

**Published:** 2018-12-04

**Authors:** Seoung Rak Lee, Yong Joo Park, Yu Bin Han, Joo Chan Lee, Seulah Lee, Hyun-Ju Park, Hae-Jeung Lee, Ki Hyun Kim

**Affiliations:** 1 School of Pharmacy, Sungkyunkwan University, Suwon 16419, Korea; davidseoungrak@gmail.com (S.R.L.); pyj084@msn.com (Y.J.P.); youbin1158@hanmail.net (Y.B.H.); leejc3004@gmail.com (J.C.L.); sarahlee0801@gmail.com (S.L.); hyunju85@skku.edu (H.-J.P.); 2 Department of Food and Nutrition, Gachon University, Seongnam 13120, Korea

**Keywords:** *Acer tegmentosum*, phenolic compounds, 1,4-benzodioxane scaffold, estrogen, phytoestrogens, molecular docking

## Abstract

Phytoestrogens derived from plants have attracted the attention of the general public and the medical community due to their potentially beneficial role in relieving menopausal symptoms. The deciduous tree *Acer tegmentosum* Maxim (Aceraceae) has long been utilized in Korean folk medicine to alleviate many physiological disorders, including abscesses, surgical bleeding, and liver diseases. In order to explore structurally and/or biologically new constituents from Korean medicinal plants, a comprehensive phytochemical study was carried out on the bark of *A. tegmentosum*. One new phenolic compound with a 1,4-benzodioxane scaffold, isoamericanoic acid B (**1**), as well as with nine known phenolic compounds (**2**–**10**), were successfully isolated from the aqueous extracts of the bark of *A. tegmentosum*. A detailed analysis using 1D and 2D NMR spectroscopy, electronic circular dichroism (ECD) spectral data, and LC/MS afforded the unambiguous structural determination of all isolated compounds, including the new compound **1**. In addition, compounds **2**, **4**, **5**, and **9** were isolated and identified from the bark of *A. tegmentosum* for the first time. All isolated compounds were tested for their estrogenic activities using an MCF-7 BUS cell proliferation assay, which revealed that compounds **1**, **2**, and **10** showed moderate estrogenic activity. To study the mechanism of this estrogenic effect, a docking simulation of compound **1,** which showed the best estrogenic activity, was conducted with estrogen receptor (ER) -α and ER-β, which revealed that it interacts with the key residues of ER-α and ER-β. In addition, compound **1** had slightly higher affinity for ER-β than ER-α in the calculated Gibbs free energy for **1**:ER-α and **1**:ER-β. Thus, the present experimental evidence demonstrated that active compound **1** from *A. tegmentosum* could be a promising phytoestrogen for the development of natural estrogen supplements.

## 1. Introduction

Estrogens regulate a variety of physiological functions on organs and tissues such as reproduction, cell growth, and differentiation [1]. In addition, estrogens have crucial role in several pathological functions including cancers, neurodegeneration, metabolic disorders, cardiovascular diseases, and osteoporosis [1,2,3,4]. Phytoestrogens, sometimes referred as “dietary estrogens”, are plant-derived natural products with the ability to bind to estrogen receptors, which exhibit estrogenic effects in relieving menopausal symptoms such as hot flashes, night sweats, mood changes, depression, and nervous tension [2,3,4]. Although various phytoestrogens including flavonoids, lignans, and anthocyanins have been reported, there are continuing demands to discover more effective and safe phytoestrogens in clinical environment [5,6,7]. 

*Acer tegmentosum* Maxim (Aceraceae) is a deciduous tree widely distributed in Korea. *A. tegmentosum* has been used in Korean traditional medicine for the treatment of various hepatic disorders, including hepatitis, cirrhosis, and liver cancer [8]. Previous phytochemical investigations on this plant have reported the isolation of various secondary metabolites with useful bioactivities, such as flavonoids, lignans, and phenolic compounds [9,10]. Among the isolated compounds, phenethyl glycosides and flavonoids were reported to exhibit hepatoprotective activities [9,10]. In a recent study, fraxin isolated from *A. tegmentosum* as a coumarin glycoside, displayed potent hepatoprotective effects through antioxidant activity and the nuclear factor erythroid-derived 2-related factor 2 (Nrf2)-mediated antioxidant enzyme system [11]. In addition, antioxidant, anti-inflammation, and anti-adipogenic activities have been reported in a biological study on the extract of *A. tegmentosum* [12,13].

As an endeavor to explore structurally and/or biologically active new constituents from Korean medicinal plants [14,15,16,17,18], a phytochemical investigation was performed on the bark of *A. tegmentosum*, which led to the isolation of one new phenolic compound (**1**) with a 1,4-benzodioxane scaffold, as well as nine known phenolic compounds (**2**–**10**) from the aqueous extracts of the bark. The planar structure of the new compound (**1**) was elucidated through analysis of 1D and 2D {^1^H-^1^H correlation spectroscopy (COSY), heteronuclear single-quantum correlation spectroscopy (HSQC), and heteronuclear multiple-bond correlation spectroscopy (HMBC)} NMR data and LC/MS analysis. In addition, the absolute configuration of **1** was determined based on quantum chemical ECD calculations. The estrogenic effects of the isolated compounds were assessed by E-screen assays with MCF-7 BUS cells. Moreover, mechanism of the estrogenic effect with estrogen receptor was investigated by using docking analysis. In this paper, the isolation and structural elucidation of compounds **1**–**10** from the bark of *A. tegmentosum*, along with their estrogenic effects, were described.

## 2. Materials and Methods 

### 2.1. General Experimental Procedures 

Optical rotations were obtained using a Jasco P-1020 polarimeter (Jasco, Easton, MD, USA). IR spectra were acquired utilizing a Bruker IFS-66/S FT-IR spectrometer (Bruker, Karlsruhe, Germany). Electrospray ionization (ESI) and HR-ESI mass spectra were transcribed using a Waters Micromass Q-TofUltima ESI-TOF mass spectrometer (Waters, New York, NY, USA). Experimental ECD spectra in MeOH were acquired in a quartz cuvette with a 1-mm optical path length on a JASCO J-1500 spectropolarimeter. (Tokyo, Japan). The ECD spectrum of MeOH was used as a baseline and subtracted from the experimental spectra. NMR spectra were recorded on a Varian UNITY INOVA 800 NMR spectrometer operating at 800 MHz (^1^H) and 200 MHz (^13^C), with chemical shifts given in ppm (δ). Preparative high performance liquid chromatography (HPLC) was performed using a Waters 1525 Binary HPLC pump with a Waters 996 Photodiode Array Detector (Waters Corporation, Milford, CT, USA). Semi-preparative HPLC utilized a Shimadzu Prominence HPLC System with SPD-20A/20AV Series Prominence HPLC UV-Vis Detectors (Shimadzu, Tokyo, Japan). LC/MS analysis was performed on an Agilent 1200 Series HPLC system (Agilent Technologies, Santa Clara, CA, USA) using an analytical Kinetex column (4.6 × 100 mm, 3.5 μm), followed by a 6130 Series ESI mass spectrometer equipped with a diode array detector. Silica gel 60 (Merck, Darmstadt, Germany; 70–230 mesh and 230–400 mesh) and RP-C18 silica gel (Merck, 40–63 μm) were used for column chromatography. The packing material for molecular sieve column chromatography was Sephadex LH-20 (Pharmacia, Uppsala, Sweden). Precoated silica gel F254 plates and RP-18 F254s plates (Merck) were used for TLC. Spots were detected on TLC under UV light or by heating after spraying with anisaldehyde–sulfuric acid. 

### 2.2. Plant Material

The bark of *A. tegmentosum* was obtained from Hongcheon and Jeongseon in the Gangwon province, Korea in June 2013. The identity of the material was confirmed by one of the authors (K. H. Kim). A voucher specimen (MIMH-35) was deposited in the herbarium of the School of Pharmacy, Sungkyunkwan University, Suwon, Korea.

### 2.3. Extraction, Fractionation, and Purification Methods

The bark of *A. tegmentosum* was dried at 60 °C for 24 h and then pulverized to afford powdered *A. tegmentosum* bark (200 g). The material was extracted with distilled water (1 L) at 90 °C for 10 h and then filtered. The filtrate was concentrated in vacuo to afford resultant extracts (13.2 g). The resultant extracts were suspended in distilled water (700 mL × 3) and successively solvent-partitioned with hexane, CH_2_Cl_2_, EtOAc, and *n*-BuOH, affording 62.1 g, 32.2 g, 22.4 g, and 37.3 g fractions, respectively. The EtOAc-soluble fraction was loaded onto a Diaion HP-20 column, and fractionated with a gradient solvent system of 20%, 40%, 60%, 80%, and 100% MeOH in H_2_O (each 500 mL). Based on the results of a TLC analysis, the 80% and 100% MeOH miscible fractions were combined into one fraction. The combined fraction (4.2 g) was separated by RP-C18 silica gel (230–400 mesh) column chromatography, with elution by a gradient solvent system of MeOH-H_2_O (1:1–1:0, *v*/*v*) to afford three fractions (A–C). Fraction B (3.3 g) was subjected to silica gel (230–400 mesh) column chromatography and separated with a gradient solvent system of EtOAc-MeOH (30:1–1:1, *v*/*v*) to provide three fractions (BA–BC). Three fractions (BA1-3) were acquired from fraction BA (511 mg) by Sephadex LH-20 column chromatography eluted with 100% MeOH. Fraction BA1 (147 mg) was further fractionated by silica gel (70–230 mesh) column chromatography with a gradient solvent system of CH_2_Cl_2_-MeOH (50:1 to 1:1, *v*/*v*) to provide six subfractions (BA11-16). Subfraction BA12 (20 mg) was purified by semipreparative reversed-phase HPLC (Phenomenex Luna Phenyl-hexyl, 250 × 10.0 mm, 5 μm) eluted with 37% MeOH/H_2_O (flow rate: 2 mL/min) to afford compound **2** (3.0 mg, *t*_R_ = 50.0 min). Three subfractions (BA21–23) were obtained from fraction BA2 (187 mg) by silica gel (70-230 mesh) column chromatography with a gradient solvent system of CH_2_Cl_2_-MeOH (30:1 to 0:1, *v*/*v*). Subfraction BA22 (62 mg) was isolated by semipreparative reversed-phase HPLC (Phenomenex Luna Phenyl-hexyl, 250 × 10.0 mm, 5 μm) using 37% MeOH/H_2_O (flow rate: 2 mL/min) to provide compounds **4** (7.6 mg, *t*_R_ = 58.0 min), **7** (4.6 mg, *t*_R_ = 55.0 min), **9** (7.3 mg, *t*_R_ = 26.0 min), and **10** (1.0 mg, *t*_R_ = 20.0 min). Fraction BB (564 mg) was loaded onto a Sephadex LH-20 column and fractionated using elution with 100% MeOH to give five fractions (BB1-BB5). Four subfractions (BB41-44) were gained from fraction BB4 (135 mg) by preparative reversed-phase HPLC (Luna C-18 column, 250 mm × 21.2 mm i.d., 5 μm) with a gradient solvent system of CH_3_CN-H_2_O (1:9–1:0, *v*/*v*, flow rate: 5 mL/min). Subfraction BB41 (15 mg) was separated by semipreparative reversed-phase HPLC (Phenomenex Luna Phenyl-hexyl, 250 × 10.0 mm, 5 μm) eluted with 30% MeOH/H_2_O (flow rate: 2 mL/min) to provide compound **8** (8.1 mg, *t*_R_ = 33.0 min). Subfraction BB44 (12 mg) was purified by semipreparative reversed-phase HPLC (Phenomenex Luna Phenyl-hexyl, 250 × 10.0 mm, 5 μm) eluted with 36% MeOH/H_2_O (flow rate: 2 mL/min) to afford compound **6** (3.9 mg, *t*_R_ = 50.0 min). Fraction BB5 (84 mg) was separated using semipreparative reversed-phase HPLC (Phenomenex Luna Phenyl-hexyl, 250 × 10.0 mm, 5 μm) eluted with 32% MeOH/H_2_O (flow rate: 2 mL/min) to provide compound **5** (4.0 mg, *t*_R_ = 42.0 min). Fraction BC (928 mg) was passed through Sephadex LH-20 column chromatography using 100% MeOH to give eight subfractions (BC1-8). Compounds **1** (7.7 mg, *t*_R_ = 45.0 min) and **3** (3.8 mg, *t*_R_ = 20.0 min) were purified from subfraction BC5 (43 mg) by semipreparative reversed-phase HPLC (Phenomenex Luna Phenyl-hexyl, 250 × 10.0 mm, 5 μm) eluted with 38% MeOH/H_2_O (flow rate: 2 mL/min).

Isoamericanoic acid B (**1**): amorphous powder; [α]${}_{D}^{25}$ + 10.6 (c 0.02, MeOH); IR (KBr) ν_max_ 3454, 1781, 1439, 1206, 1054 cm^−1^; UV (MeOH) λ_max_ (log ε) 225 (3.90), 257 (3.75), 289 (3.41) nm; ECD (MeOH) λ (△ε) 206 (1.23), 220 (−7.20), 238 (0.62) nm; ^1^H (800 MHz) and ^13^C NMR (200 MHz) data, see Table 1; HR-ESIMS (negative ion-mode) *m/z* 377.0873 [M−H]^−^ (calcd for C_18_H_18_O_9_, 377.0873). 

### 2.4. Computational Analysis

To obtain the conformational differences between **1a** (7*R*,8*R*) and **1b** (7*S*,8*S*), computational discrete fourier transform (DFT) calculations were carried out. The first structural energy minimization of **1a** and **1b** were performed by utilizing the Avogadro 1.2.0 software (Pittsburgh, PA, USA) with the UFF force field. Then, the ground-state geometries of **1a** and **1b** were established using Tmolex 4.3.1 software (Leverkusen, Germany) with the DFT settings (B3-LYP functional/M3 grid size), geometry optimization options (energy 10^−6^ hartree, gradient norm |dE/dxyz| = 10^−3^ hartree/bohr), and the basis set def-SV(P) for all atoms. The calculated ECD spectra of the optimized structures of **1a** and **1b** were acquired using the B3LYP/DFT functional settings, with the basis set def2-TZVPP, for all atoms. The obtained CD spectra were simulated by overlying each transition, where *σ* is the width of the band at 1/e height. ΔE_i_ and R_i_ are the excitation energies and rotatory strengths for transition i, respectively. In the present study, the value of σ was 0.10 eV.

$$\Delta \epsilon \left(E\right)=\frac{1}{2.297\times 10^{-39}}\frac{1}{\sqrt{2\pi \sigma }}\sum_{A}^{i}\Delta E_{i}R_{i}e^{[-{\left(E-\Delta E_{i}\right)}^{2}/\left(2\sigma {)}^{2}\right]}$$

### 2.5. Cell Culture and WST-1 Cell Cytotoxicity Assays

Estrogen-sensitive MCF-7 BUS human breast cancer cells were kindly provided by Dr. Soto (Tufts University, Boston, MA, USA). MCF-7 BUS cells were maintained in red-dulbecco’s modified eagle medium (DMEM) supplemented with 5% fetal bovin serum (FBS), penicillin (100 units/mL), and streptomycin (100 μg/mL) in a humidified incubator at 37 °C and 5% CO_2_/95% air. The cellular cytotoxicity of compounds was tested by WST-1 assays. Briefly, cells were seeded at 5000 cells/100 µl medium/well into 96-well plates. After 24 h of incubation, the medium was replaced with experimental charcoal dextran-treated medium (10% CD-FBS supplemented with phenol red-free DMEM) containing test compounds at various concentrations. Cells were incubated with test compounds for 48 h, after which WST-1 reagent was added to each well (final dilution, 1:10). Cells were then incubated for 1 h at 37 °C. Cell viability was quantified by measuring the absorbance at 440 nm using a VERSAmax microplate reader (Molecular Devices, Sunnyvale, CA, USA).

### 2.6. E-Screen Assay

The estrogenic effects of compounds were assessed by E-screen assays with MCF-7 BUS cells, as previously described [19]. Briefly, the cells were harvested and suspended in 5% FBS DMEM before being seeded into 48-well plates at 5 × 10^3^ cells/well. After 48 h of incubation, the medium was replaced with charcoal dextran-treated medium containing test compounds at various concentrations. After a further 144 h of incubation, cellular proliferation was measured using the sulforhodamine B staining method. Cellular proliferation data are represented as relative proliferative effects (RPEs) of MCF-7 BUS cells, which were calculated as follows: RPE = {(S − 1)/(E − 1)} × 100, where S = proliferation of experimental samples and E = proliferation of positive control (10^−9^ M E_2_).

### 2.7. RT-PCR Assay

MCF-7 BUS cells were seeded into a six-well plate and incubated for 24 h at 37 °C. The medium was replaced with charcoal dextran-treated medium containing E_2_ (10^−9^ M) or compound **1** (80 µM). After 48 h of incubation, total RNA was extracted using RNAiso Plus reagent (Takara, Shiga, Japan). Complementary DNA (cDNA) was synthesized by using a RT Premix kit (Bioneer, Daejeon, Korea) with 0.5 μg/mL random primers. The PCR was performed in PCR thermocycler (MJ Ressearch; Marshall Scientific, Hampton, NH, USA). The PCR amplification for pS2 and α-actin was 29 cycles at 94 °C for 30 s, 55 °C for 1 min, 72 °C for 2 min. The PCR product run in 1.5% agarose gel electrophoresis in TBE buffer with ethidium bromide. The PCR products were detected and analyzed with gel documentation and analysis system (UVP).

(1) α-actin Forward: 5′-GGAGCAATGATCTTGATCTT-3′
(2) α-actin Reverse: 5′-CCTTCTGGGCATGGAGTCCT-3′
(3) pS2 Forward: 5′-CATGGAGAACAAGGTGATCTG-3′
(4) pS2 Reverse: 5′-CAGAAGCGTGTCTGAGGTGTC-3′

### 2.8. Molecular Docking

Docking modeling was carried out using the molecular modeling package Sybyl-X 2.1.1 (Tripos Inc, St Louis, MO, USA). Structure of **1** was sketched using sketch module, and energy minimization was conducted under the Tripos force field using the conjugated gradient method, which was set to converge to the maximum derivatives of 0.001 kcal mol^−1^.Å^−1^. To prepare the receptor protein, the coordinate files of estrogen receptor (ER)-α (PDB id: 1A52) [20] and ER-β (PDB id: 5TOA) [21] were retrieved from the Protein Data Bank (PDB). All water molecules and duplicated chains were removed, and the bound 17β-estradiol was extracted. The extracted 17β-estradiol was also prepared for re-docking by adding hydrogen atoms, correcting atom types and wrong valences, and assigning Gasteiger-Hückel charge on all atoms. Each protomol of ER was generated based on the native ligand, 17β-estradiol. For docking process, all parameters were set to default, and 50 conformers per ligand were generated. The binding affinities of each ligand docking pose were calculated by Surflex-Dock scores and the consensus scoring function. The high-ranked poses with good Surflex-Dock scores (total score, Cscore) were selected, and the representative docking model was selected by visual inspection by considering the important binding interactions of compound **1** with the receptor. 

### 2.9. Statistical Analysis

GraphPad Prism software (GraphPad Software, La Jolla, CA, USA) and Excel 2016 (Microsoft, Redmond, WA, USA) were used for data analysis. Each in vitro assay was performed at least in triplicate, and the data from each assay were expressed as means ± standard deviations (SDs). Statistical significance was assessed by a one-way analysis of variance (ANOVA) followed by Duncan’s post hoc test. Differences with *p* < 0.05 were considered statistically significant.

## 3. Results and Discussion

### 3.1. Phytochemical Investigation and Isolation of Compounds 

**Figure 1:**
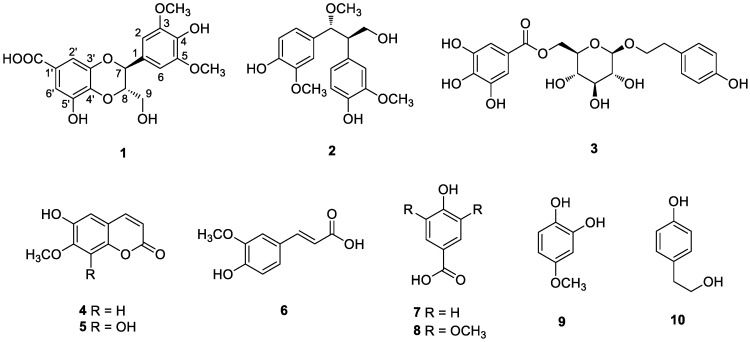
Chemical structures of compounds **1**–**10**.

Dried and pulverized *A. tegmentosum* bark was extracted with distilled water at 90 °C for 10 h and then filtered. The filtrate was evaporated under reduced pressure with a rotavapor to obtain a crude aqueous extract. The resultant extracts were suspended in distilled water and successively solvent-partitioned with hexane, CH_2_Cl_2_, EtOAc, and *n*-BuOH, affording four main fractions. According to an LC/MS analysis equipped with in-house UV spectra library, the EtOAc-soluble fraction was determined to contain promising compounds, which led us to investigate the EtOAc-soluble fraction to identify structurally and/or biologically active new constituents. Extensive, repeated column chromatography and semi-preparative HPLC purification of the EtOAc-soluble fraction afforded the isolation of one new phenolic compound (**1**) with a 1,4-benzodioxane scaffold, together with nine known phenolic compounds (**2**–**10**) (Figure 1).

### 3.2. Structural Identification of the Isolated Compounds 

**Figure 2:**
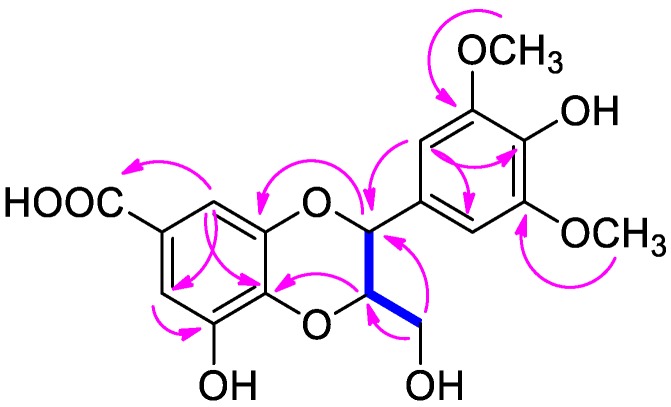
Key ^1^H-^1^H COSY (blue lines) and key HMBC (heteronuclear multiple-bond correlation spectroscopy) (pink arrows) for compound **1**.

Compound **1** was purified as an amorphous powder with a positive specific rotation value ([α]${}_{D}^{25}$ + 10.6, in MeOH), and its molecular formula was determined to be C_18_H_18_O_9_ from the [M–H]^−^ peak at *m/z* 377.0873 (calcd for C_18_H_18_O_9_, 377.0873) in the HR-ESIMS data. The ^1^H NMR spectrum of compound **1** (Table 1) displayed signals for aromatic protons at *δ*_H_ 6.64 (2H, s), and 7.04 (2H, br s); two oxygenated methines at *δ*_H_ 4.07 (1H, m) and 4.75 (1H, d, *J* = 8.0 Hz); one oxygenated methylene at *δ*_H_ 3.47 (1H, dd, *J* = 12.0, 6.0 Hz) and 3.56 (1H, d, *J* = 12.0 Hz); and two methoxy groups at *δ*_H_ 3.77 (6H, s). The ^13^C NMR data of **1** (Table 1) showed a total of 18 carbon resonances attributable to 12 aromatic sp^2^ carbons at *δ*_C_ 106.1 (×2), 111.3, 111.4, 125.5, 128.3, 137.5, 137.6, 145.7, 147.3, and 149.7 (×2); three oxygenated carbons at *δ*_C_ 62.3, 78.2, and 80.4; two methoxy carbons at *δ*_C_ 57.0 (×2); and one ester carbon at *δ*_C_ 170.9. Thorough analysis of the ^1^H and ^13^C NMR data of **1** revealed that the NMR data were similar to those of 3-*O*-methyl isoamericanoic acid A, identified from the bark of *Picea jezoensis*, with apparent differences being that the signals for aromatic protons were absent in 3-*O*-methyl isoamericanoic acid A and the signals for an additional methoxy group [*δ*_H_ 3.77 (s); *δ*_C_ 57.0] were present in compound **1** [22]. The complete structure of **1** based on the inspection of the above ^1^H and ^13^C NMR data was further confirmed by the interpretation of 2D NMR experiments (^1^H-^1^H COSY, HSQC, and HMBC) (Figure 2). The position of the additional methoxy group was determined to be C-5 based on an HMBC correlation from the methoxy protons to C-5, as well as the presence of symmetric protons and carbons [*δ*_H_ 6.64 (s); *δ*_C_ 106.1]. The down-field shifted quaternary carbon at *δ*_C_ 147.3 (C-5′) and HMBC correlations from H-6′ to C-1′, C-2′, C-4′, and C-5′ suggested that a hydroxyl group was substituted at C-5′.

**Figure 3:**
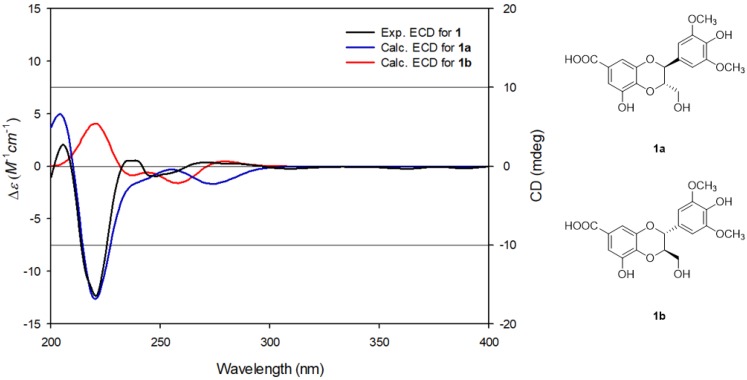
Experimental and calculated ECD (electronic circular dichroism) spectra of compounds **1**, **1a**, and **1b**.

The relative stereochemistry of **1** was deduced by utilizing the *J*-based configuration analysis (JBCA) method. The coupling constant of *J*_H7,H8_ = 8.0 Hz in the ^1^H NMR data revealed that the relative configuration between H-7 and H-8 was *trans* [23,24]. The absolute configuration of **1** was determined by comparing the experimental ECD spectrum of **1** with calculated ECD data of **1a** (7*S*,8*S*) and **1b** (7*R*,8*R*). The experimental ECD spectrum of **1** exhibited a negative Cotton effect at around 220 nm, matching well with the calculated ECD data for **1a** (Figure 3). Thus, the absolute configuration of C-7 and C-8 in **1** was assigned as 7*S*, 8*S*, respectively. Accordingly, the chemical structure of **1** was finally determined, as shown in Figure 1, and named as isoamericanoic acid B. Recently, a pair of enantiomeric methyl esters [(7*S*,8S)- and (7*R*,8*R*)-pithecellobiumin A] of compound **1** was isolated from the twigs and leaves of *Pithecellobium clypearia* [25] and exhibited anti-A*β* aggregation activity.

The known compounds were identified as 4,4′-((1*R*,2*R*)-3-hydroxy-1-methoxypropane-1,2-diyl)bis(2-methoxyphenol) (**2**) [26], 6-*O*-galloylsalidroside (**3**) [27], isoscopoletin (**4**) [28], 6,8-dihydroxy-7-methoxy-2*H*-1-benzopyran-2-one (**5**) [29], *trans*-ferulic acid (**6**) [30], 4-hydroxybenzoic acid (**7**) [31], syringic acid (**8**) [32], 4-methoxycatechol (**9**) [33], and 4-hydroxyphenylethanol (**10**) [34] by comparing the spectroscopic data obtained in this study with values reported in previous studies. To the best of our knowledge, compounds **2**, **4**, **5**, and **9** were structurally identified from the bark of *A. tegmentosum* for the first time in this study.

### 3.3. Estrogenicity of Compounds Isolated from A. Tegmentosum

During the menopausal transition, many women suffer menopausal symptoms, such as hot flashes, night sweats, mood changes, depression and nervous tension, due to lower estrogen levels [35,36]. These symptoms can be ameliorated by hormone replacement therapy (HRT). However, current HRT appears to be associated with increased risks of developing breast and ovarian cancers in healthy women and have limitations to use for patients with breast cancer [37]. To overcome the shortcomings of HRT, new and safe phytoestrogens, which are plant-derived compounds resembling estrogen, have emerged as an alternative to conventional HRT to alleviate the symptoms of menopause [38]. Phytoestrogens are potential HRT agents that can act as both estrogen and anti-estrogen agents depending on the circulating endogenous estrogen levels [36]. Phytoestrogens mainly belong to a large group of natural phenolic compounds and they are known to exert their effects primarily through binding to estrogen receptors (ER), ER-α, and ER-β. ER-α is present mainly in female reproductive tissues (uterus and ovary), breast, kidney, bone, lung, and cardiovascular system, while ER-β is found in all over the body regardless of sex [1,2,5]. Among phytoestrogens, isoflavones are considered to be the most active natural products with respect to estrogenic effects. 

**Figure 4:**
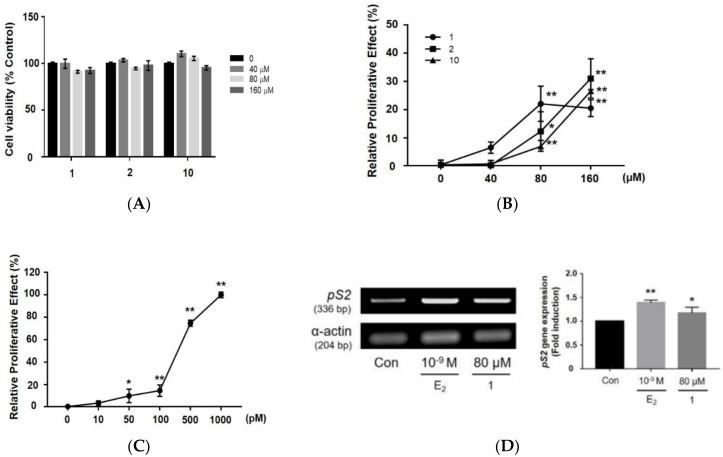
Cytotoxicity and estrogenic effects of compounds **1**, **2**, and **10,** isolated from *Acer tegmentosum*, on MCF-7 BUS cells. (**A**) The effects of the compounds on MCF-7 BUS cell viability were tested by WST-1 assays. Cells were treated with each compound at 20 to 160 µM for 48 h, and cell viability was quantified with absorbance at 440 nm using a microplate reader. Estrogenic effects of (**B**) compounds (40 to 160 µM) and (**C**) 17β-estradiol (E_2_; 10 -1000 pM) were measured by E-screen assays. Cells were treated with the indicated concentrations of the compounds for 144 h, then sulforhodamine B (SRB) assays were conducted to measure cell proliferation. Cell proliferation relative to that in the presence of E_2_ (10^−9^ M; 1000 pM, 100%) was represented as the relative proliferative effect and expressed as mean ± SD of three separate experiments for each group. (**D**) Effects of compound **1** on pS2 mRNA expression. Cells were treated with compound **1** for 48 h, then total mRNA was extracted using RNAiso Plus reagent. pS2 mRNA levels were measured by RT-PCR and normalized using α-actin mRNA as an internal standard. Symbols (*, **) represent statistical differences from the vehicle control (Con, 0.1% DMSO): *p < 0.05, **p < 0.01.

First, the cytotoxicity of the compounds isolated from *A. tegmentosum* were tested using a WST-1 assay after 48 h of treatment (Figure 4A). Compounds that were not cytotoxic (at 40, 80, and 160 µM) were selected for assessment of their estrogenic activity. Compounds were dispersed in CD-FBS medium and incubated with MCF-7 BUS cells for 144 h. The estrogenicity of the compounds was calculated as the cell proliferation relative to that seen in the presence of 10^−9^ M 17β-estradiol and is represented as the relative proliferative effect (RPE). Among the isolated compounds, compounds **1**, **2**, and **10** showed estrogenic activities (Figure 4B). At 80 µM, each compound showed 22.1 (**1**), 12.3 (**2**), and 6.9 (**10**) RPE values, which is similar to the pattern observed with 163, 69 and 17 pM of 17β-estradiol (Figure 4C). In addition, *pS2* gene expression was measured after treatment of compound **1**, which showed the highest estrogen activity at 80 µM. The *pS2* gene is regulated by estrogen at the transcriptional level and considered ideal marker to measure the estrogen activity [39]. Compound **1** induced pS2 gene expression (Figure 4D), suggesting that compound **1** can be a phytoestrogen with moderate estrogenic activity.

### 3.4. Molecular Docking of Compound **1** into ER-α and ER-β

**Figure 5:**
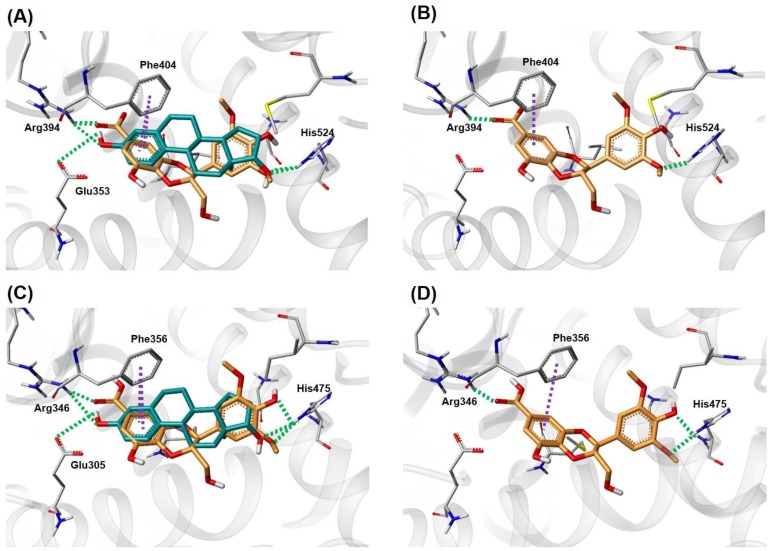
Proposed binding pose of compound **1** in the active site of ER-α (PDB id: 1A52) and ER-β (PDB id: 5TOA). The docked pose of **1** and the co-crystalized pose of 17β-estradiol were superimposed in the active site of ER-α (**A**) and ER-β (**C**). All atoms of the ligands and amino acid residues are colored by atom type: carbon atoms are colored green-blue (17β-estradiol), orange (**1**), and grey (amino acid residues); nitrogen is blue; oxygen is red; sulfur is yellow; and hydrogen is white. The hydrogen bonds are indicated as green dashes and π-π interactions as purple dashes. The docked pose of **1** in the ligand-binding pocket of ER-α (**B**) and ER-β (**D**).

In the E-screen assay results, compound **1** showed the best estrogenic effect in MCF-7 BUS cells among the isolates in this study. Additionally, compound **1** has a similar shape to genistein, a well-known natural product ER agonist, with 73.4% similarity (calculated by the Tanimoto coefficient using the MACCS fingerprint in KNIME 3.6.0 (KNIME AG, Zurich, Switzerland)). To examine the potential binding pose of **1** in the active site of ER, a docking analysis of **1** using X-ray structures of ER-α (PDB id: 1A52) [20] and ER-β (PDB id: 5TOA) [21] in complex with 17β-estradiol was carried out. As illustrated in Figure 5, compound **1** fit into the ligand-binding pocket of ER and overlaid well atop the X-ray pose of 17β-estradiol. The key interactions between most ER agonists and ER are: (1) hydrogen bonds (H-bonds) with polar residues where two hydroxyl groups in the A and D ring of 17β-estradiol are anchored by forming H-bonds, and (2) hydrophobic interactions with the amino acid residues in the steroid ring binding pocket. For compound **1**, the carboxyl substituent attached to benzodioxane group formed H-bonds with Arg394 (ER-α) and Arg346 (ER-β), and the phenolic OH and the methoxy oxygen atom on 2,6-dimethoxyphenol ring were H-bonded to His524 (ER-α) and His475 (ER-β). In addition, the benzene ring of the benzodioxane group interacted with Phe404 (ER-α) and Phe356 (ER-β) by a π-π stacking interaction inside the hydrophobic pocket. The same interactions were also revealed in the X-ray structures, in which the aromatic A ring of 17β-estradiol formed a π-π stacking interaction with Phe404 (ER-α) and Phe356 (ER-β). These docking results suggest the capability of compound **1** to bind to the ligand-binding pocket of both ER-α and ER-β and act as a phytoestrogen. The binding affinity of **1** was also estimated in comparison with 17β-estradiol using the Surflex-Dock score (−log*K_d_*). The scores for 17β-estradiol were obtained by re-docking of the crystallographic binding pose extracted from ER-α and ER-β, respectively. (Figure S7 in Supplementary Materials) The calculated Gibbs free energy for **1**:ER-α, **1**:ER-β, 17β-estradiol:ER-α, and the 17β-estradiol:ER-β complex are −2.36, −2.69, −7.21, and −5.63 kcal/mol, respectively (Table 2). The results suggest that the **1**:ER complex is less stable than 17β-estradiol:ER in the equilibrium, which is consistent with the estrogenic activity difference for **1** and 17β-estradiol. The results from estimated binding affinity indicated that compound **1** exhibits estrogenic activity and slightly higher binding affinity for ER-β than for ER-α. Activation of ER-β is known to lead inhibition of cell proliferation, tumor suppression, and protection of atherosclerosis [40,41]. Phytoestrogens tend to have both estrogenic and anti-estrogenic effects depending on the circulating estrogen levels when they have higher binding affinity for ER-β than for ER-α [42]. After menopause, estrogen levels start to decline, and menopause symptoms are often troublesome to women [35,43]. Thus, these results suggest that compound **1** may be useful as a promising phytoestrogen for the development of natural estrogen supplements.

## 4. Conclusions

In the present study, the therapeutic potential of the isolated phenolic compounds (**1**–**10**) as phytoestrogens was investigated, where compound **1** was a newly isolated natural product, isoamericanoic acid B. In the E-screen assays, compounds **1**, **2**, and **10** displayed the estrogenic activities with 22.1 (**1**), 12.3 (**2**), and 6.9 (**10**) RPE values, respectively, suggesting that they may be useful as promising phytoestrogens for natural estrogen supplements. Additionally, the docking simulation results of compound **1**, which exhibited the best estrogenic effects in MCF-7 BUS cells, showed interactions between compound **1** and the key residues of ER-α (Arg394, His524, Phe404) and ER-β (Arg346, His475, Phe356) in their active binding sites. Compound **1** also exhibited slightly higher affinity for ER-β than ER-α in the calculated Gibbs free energy for **1**:ER-α and **1**:ER-β. These results demonstrate that compound **1** from the bark of *A. tegmentosum* could be a promising phytoestrogen to alleviate menopausal symptoms.

## Figures and Tables

**Table 1:** ^1^H (800 MHz) and ^13^C (200 MHz) nuclear magnetic resonance (NMR) data of **1** in CD_3_OD ^a^.

Position	1	
	*δ*_C_	*δ*_H_ (*J* in Hz)
1	128.3 s	
2,6	106.1 d	6.64 s
3,5	149.7 s	
4	137.6 s	
7	78.2 d	4.75 d (8.0)
8	80.4 d	4.07 m
9	62.3 t	3.47 dd (12.0, 6.0)
		3.56 d (12.0)
1′	125.5 s	
2′	111.3 d	7.04 br s
3′	145.7 s	
4′	137.5 s	
5′	147.3 s	
6′	111.4 d	7.04 br s
3,5-OMe	57.0 q	3.77 s
1′-COOH	170.9 s	

^a^ Coupling constants (in parentheses) are in Hz.

**Table 2:** Calculated Gibbs free binding energy for **1**:ER versus the 17β-estradiol:ER complex.

ER-α (pdb id: 1A52)				ER-β (pdb id: 5TOA)			
Ligand	Surflex-Dock Score (−log*K_d_*)	*K_d_*	Gibbs Free Energy * (Δ*G_bind_*)	Ligand	Surflex-Dock Score (−logK_d_)	*K_d_*	Gibbs Free Energy * (Δ*G_bind_*)
**1**	1.7299	1.86 × 10^−2^	−2.36 kcal/mol	**1**	1.9724	1.06 × 10^−2^	−2.69 kcal/mol
**17β-estradiol**	5.2866	5.15 × 10^−6^	−7.21 kcal/mol	**17β-estradiol**	4.1318	7.38 × 10^−5^	−5.63 kcal/mol

* Δ*G_bind_* = *RTlnK_d_* (R = 1.987 cal/moL K, T = 298 K).

## Supplementary Materials

The following are available online at http://www.mdpi.com/2072-6643/10/12/1915/s1, Figure S1: The HR-ESIMS data of **1**, Figure S2: The 1H NMR spectrum of **1** (CD3OD, 800 MHz). Figure S3: The 13C NMR spectrum of **1** (CD3OD, 200 MHz). Figure S4: The 1H-1H COSY spectrum of 1 (CD3OD). Figure S5: The HSQC spectrum of **1** (CD3OD). Figure S6: The HMBC spectrum of **1** (CD_3_OD). Figure S7: Re-docked pose of 17β-estradiol in the active site of ER-α and ER-β. Figure S8: Calculated Gibbs free binding energy for **1** and 17β-estradiol. Figure S9: The ^1^H NMR spectrum of **2** (CD_3_OD, 800 MHz). Figure S10: The ^1^H NMR spectrum of **3** (CD_3_OD, 800 MHz). Figure S11: The ^1^H NMR spectrum of **4** (CD_3_OD, 800 MHz). Figure S12: The ^1^H NMR spectrum of **5** (CD_3_OD, 800 MHz). Figure S13: The ^1^H NMR spectrum of **6** (CD_3_OD, 800 MHz). Figure S14: The ^1^H NMR spectrum of **7** (CD_3_OD, 800 MHz). Figure S15: The ^1^H NMR spectrum of **8** (CD_3_OD, 800 MHz). Figure S16: The ^1^H NMR spectrum of **9** (CD_3_OD, 800 MHz). Figure S17: The ^1^H NMR spectrum of **10** (CD_3_OD, 800 MHz).
