# Supplementary material for: Isoamericanoic Acid B from Acer tegmentosum as a Potential Phytoestrogen
Source: Nutrients. 2018 Dec 4;10(12):1915. doi: 10.3390/nu10121915 (PMC6315828; doi:10.3390/nu10121915)
Supplement: Supplementary file 1 [file nutrients-10-01915-s001.doc]

**Supplementary Materials**

Isoamericanoic Acid B from *Acer tegmentosum* as A Potential Phytoestrogen

Seoung Rak Lee ^1^, Yong Joo Park ^1^, Yu Bin Han ^1^, Joo Chan Lee ^1^, Seulah Lee ^1^, Hyun-Ju Park ^1^, Hae-Jeung Lee ^2^,*, and Ki Hyun Kim ^1^,*

^1^ School of Pharmacy, Sungkyunkwan University, Suwon 440–746, Korea

^2^ Department of Food and Nutrition, Gachon University, Seongnam 13120, Korea

**Supporting Information Contents:**

**Figure S1.** The HR-ESIMS data of **1**………………..……………………………………………………………………………………….S3

**Figure S2.** The ^1^H NMR spectrum of **1** (CD_3_OD, 800 MHz)...…………………………………………………………………………….S4

**Figure S3.** The ^13^C NMR spectrum of **1** (CD_3_OD, 200 MHz)..………………………………….…………………….……………………S5

**Figure S4.** The ^1^H-^1^H COSY spectrum of **1** (CD_3_OD)..………………………………………….…………………….……………………S6

**Figure S5.** The HSQC spectrum of **1** (CD_3_OD)….…………………………………………………………………………………………S7

**Figure S6.** The HMBC spectrum of **1** (CD_3_OD)……………………………………………………………………………………………S8

**Figure S7.** Re-docked pose of 17β-estradiol in the active site of ER-α and ER-β………………………………………………………S9

**Figure S8.** Calculated Gibbs free binding energy for **1** and 17β-estradiol……………………………………………………………S10

**Figure S9.** The ^1^H NMR spectrum of **2** (CD_3_OD, 800 MHz)...………………………………………………………………………….S11

**Figure S10.** The ^1^H NMR spectrum of **3** (CD_3_OD, 800 MHz)...………………………………………………………………………….S12

**Figure S11.** The ^1^H NMR spectrum of **4** (CD_3_OD, 800 MHz)...………………………………………………………………………….S13

**Figure S12.** The ^1^H NMR spectrum of **5** (CD_3_OD, 800 MHz)...………………………………………………………………………….S14

**Figure S13.** The ^1^H NMR spectrum of **6** (CD_3_OD, 800 MHz)...………………………………………………………………………….S15

**Figure S14.** The ^1^H NMR spectrum of **7** (CD_3_OD, 800 MHz)...………………………………………………………………………….S16

**Figure S15.** The ^1^H NMR spectrum of **8** (CD_3_OD, 800 MHz)...………………………………………………………………………….S17

**Figure S16.** The ^1^H NMR spectrum of **9** (CD_3_OD, 800 MHz)...………………………………………………………………………….S18

**Figure S17.** The ^1^H NMR spectrum of **10** (CD_3_OD, 800 MHz)...……………………………………………………………………….S19


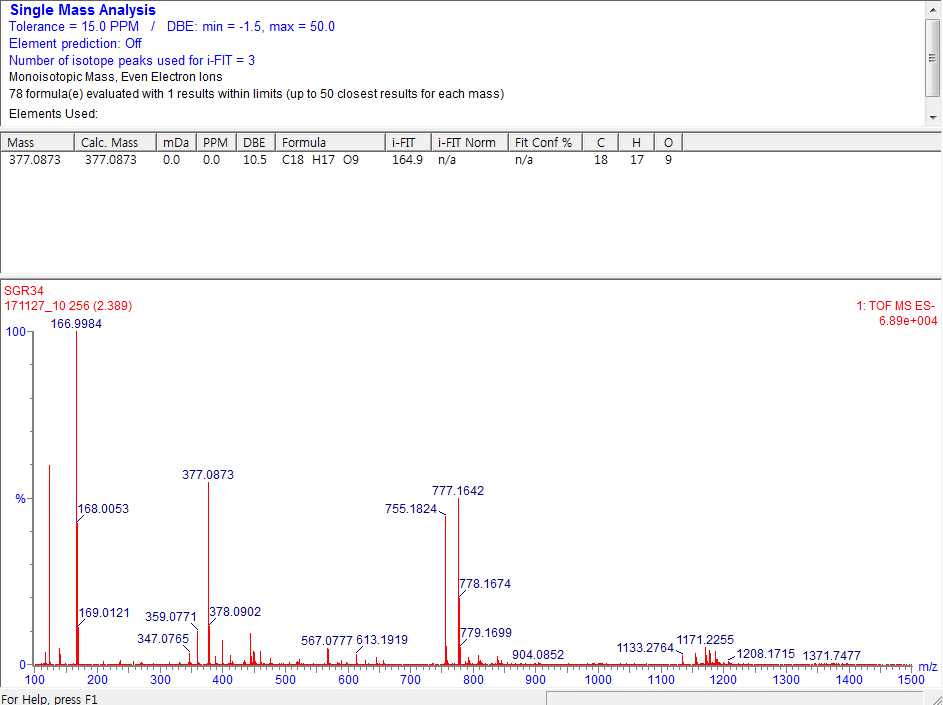


[M-H]^-^

**Figure S1.** The HR-ESIMS data of **1.**


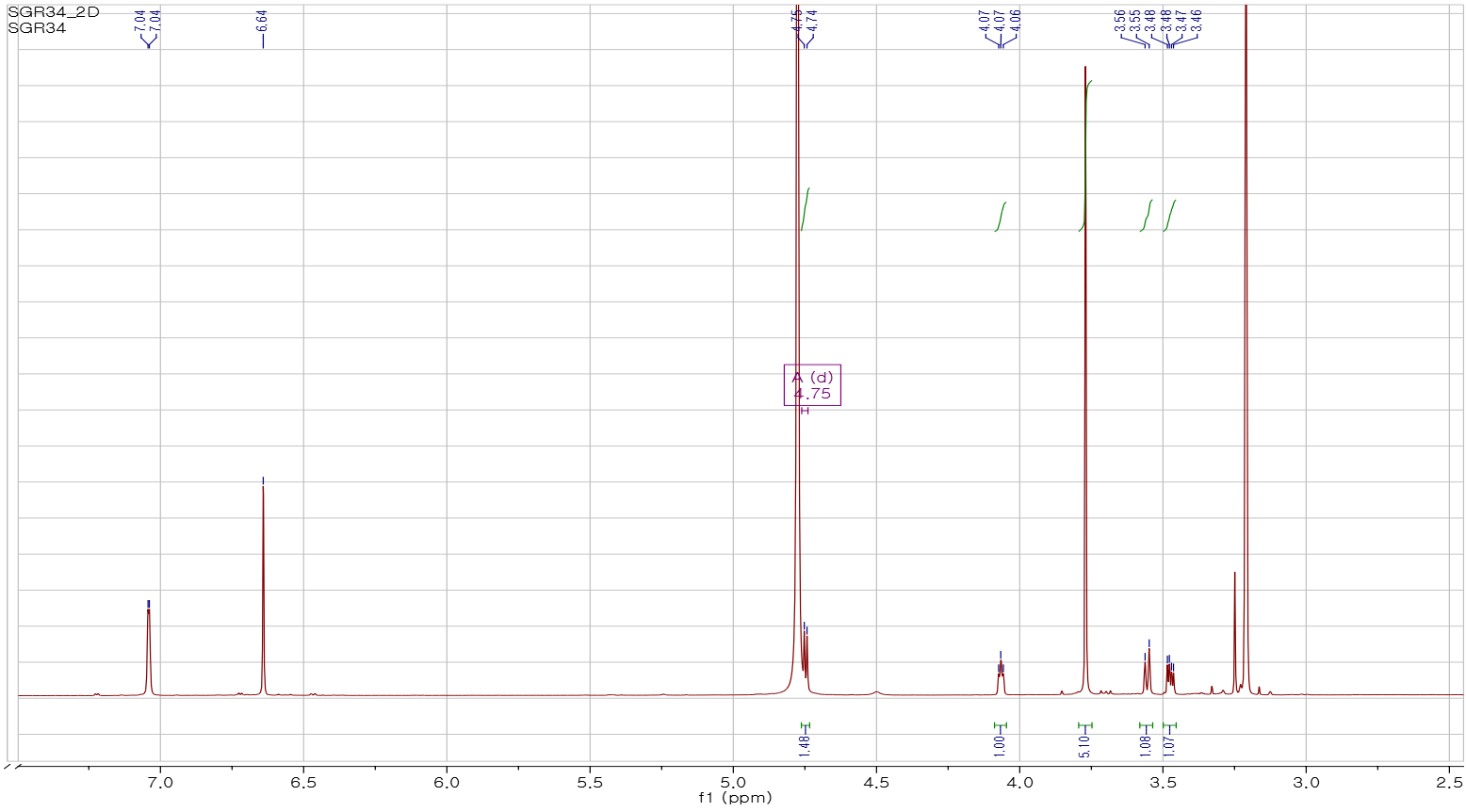


**Figure S2.** The ^1^H NMR spectrum of **1** (CD_3_OD, 800 MHz).


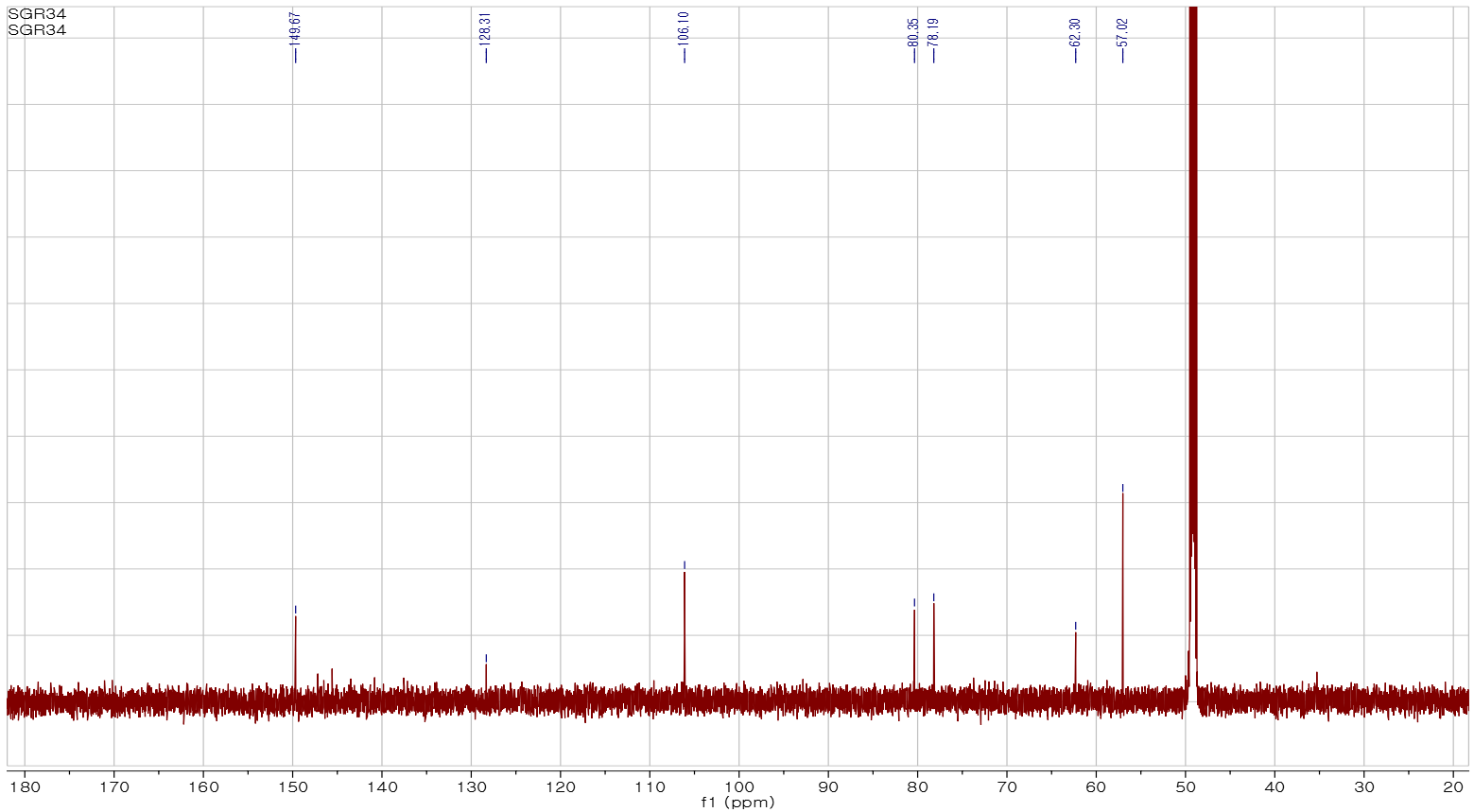


**Figure S3.** The ^13^C NMR spectrum of **1** (CD_3_OD, 200 MHz).


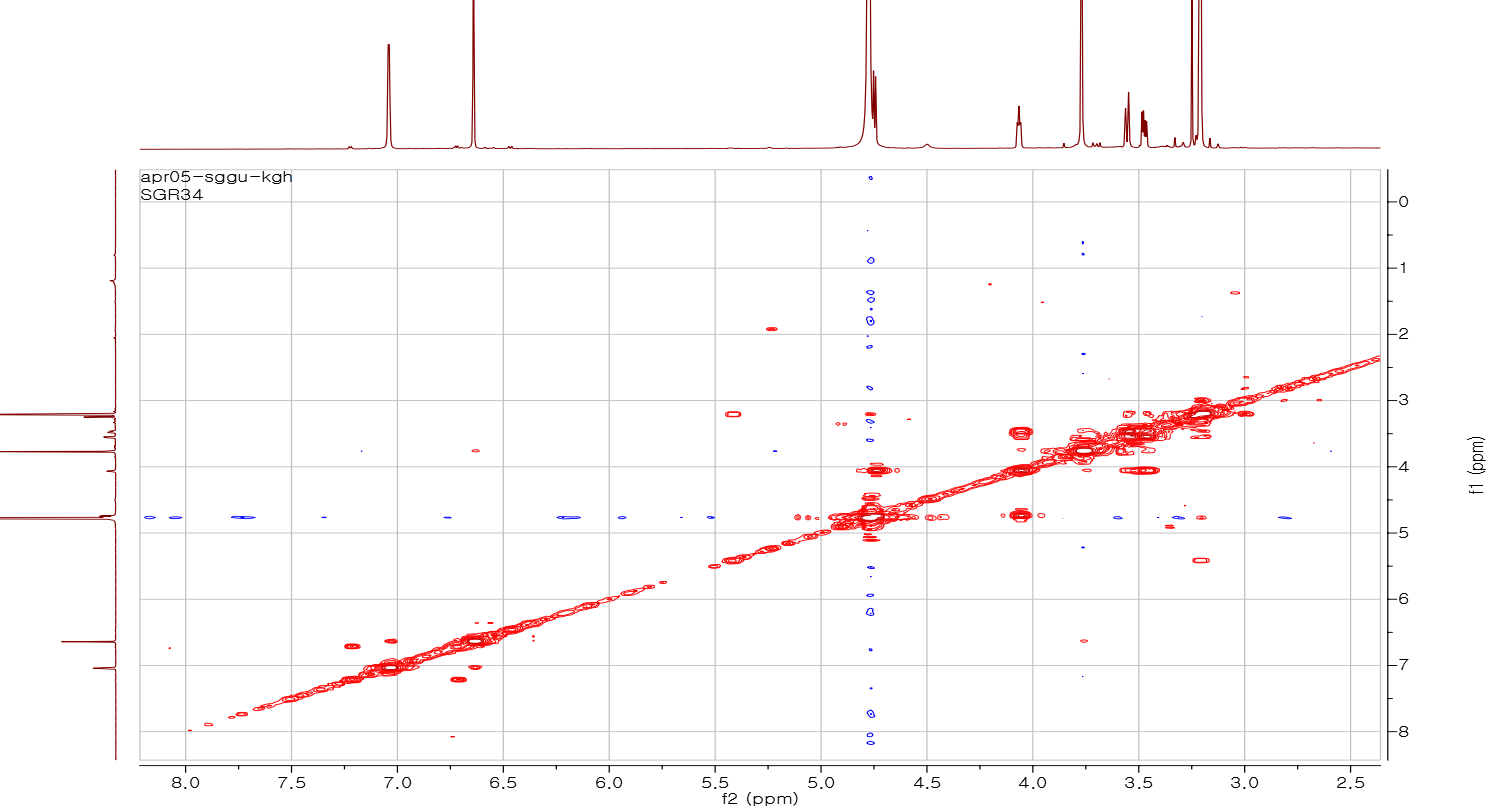


**Figure S4.** The ^1^H-^1^H COSY spectrum of **1** (CD_3_OD).


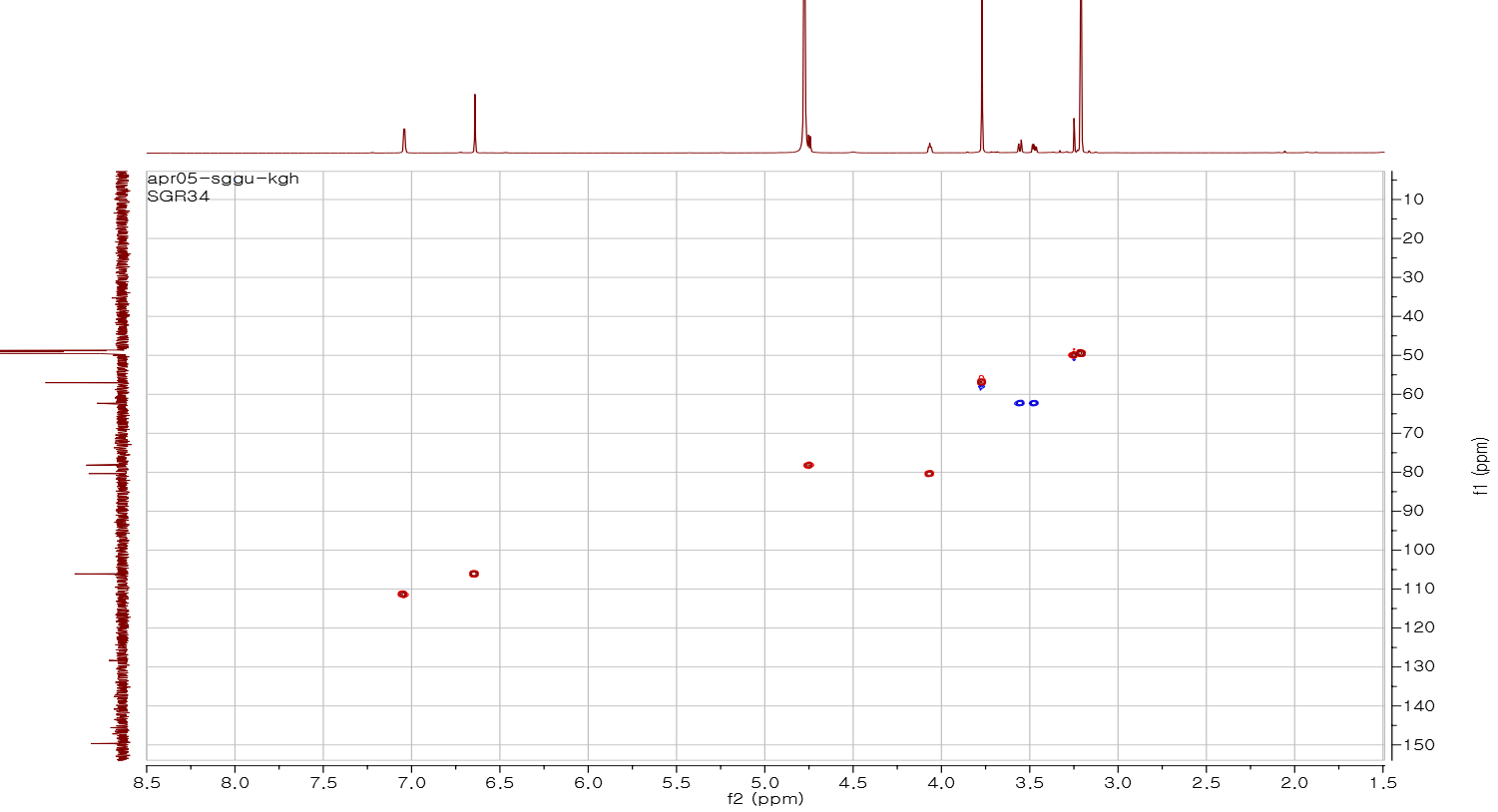


**Figure S5.** The HSQC spectrum of **1** (CD_3_OD).


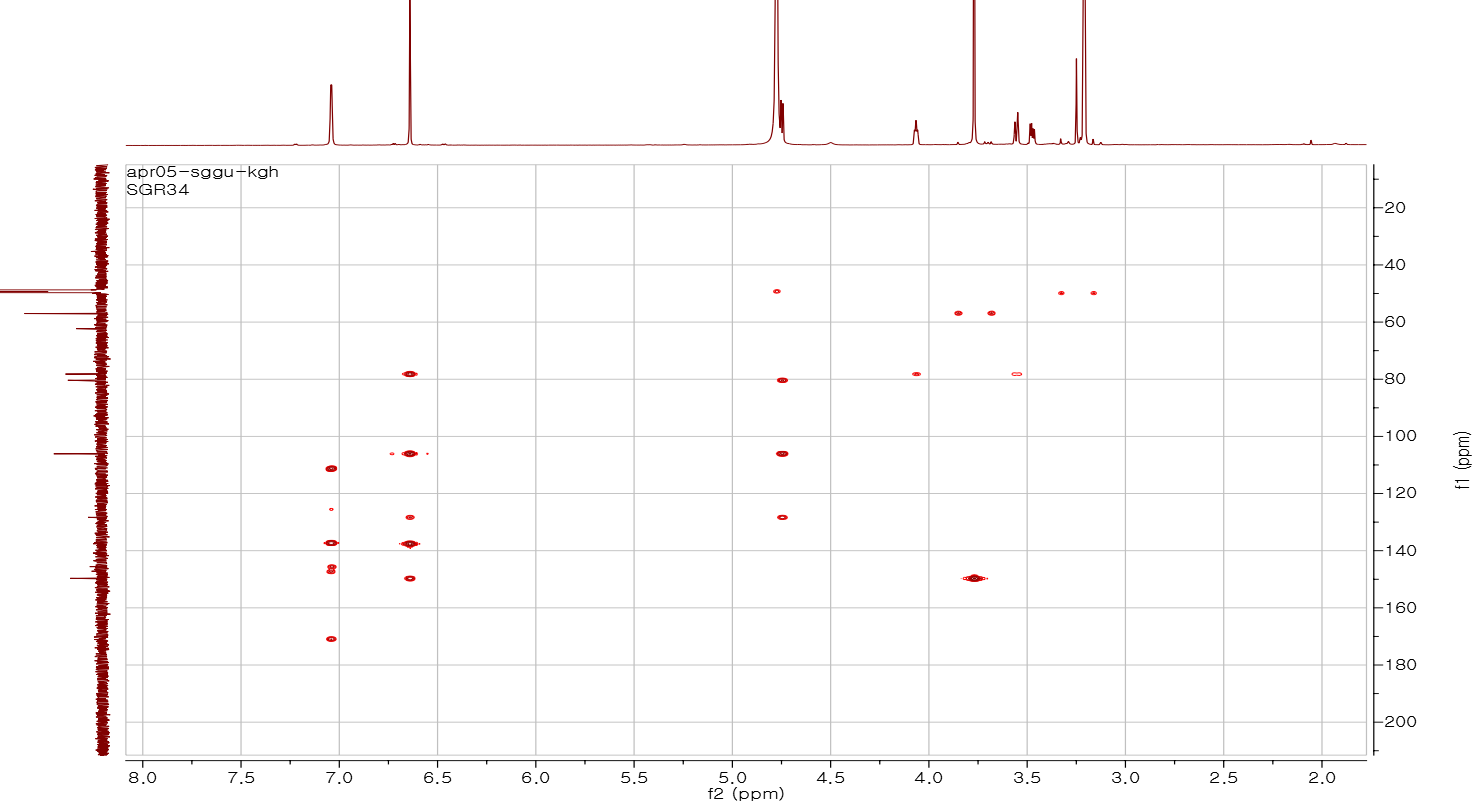


**Figure S6.** The HMBC spectrum of **1** (CD_3_OD).

**
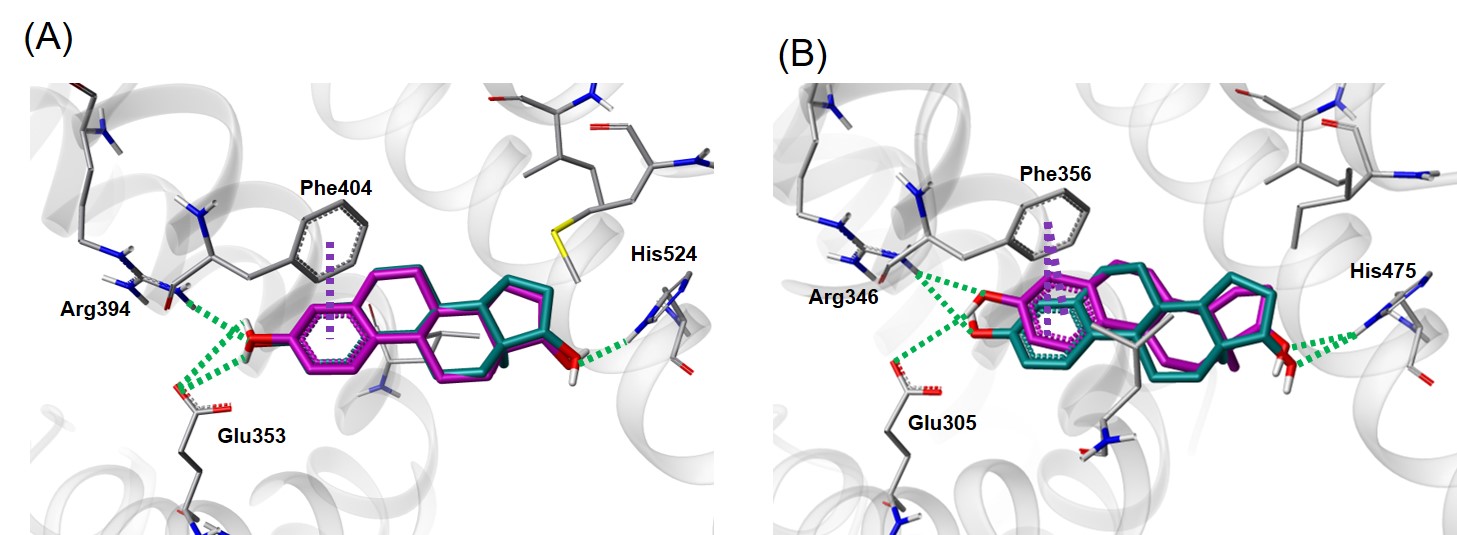
**

**Figure S7.** Re-docked pose of 17β-estradiol superimposed over X-ray pose in the active site of (**A**) ER-α (PDB code: 1A52) and (**B**) ER-β (PDB code: 5TOA). Carbon is green blue (re-docked), magenta (co-crystalized) and grey (amino acid residues). Other atoms are colored by atom type. The hydrogen bonds are presented as green dashes and π-π interaction as purple dashes, respectively.

**
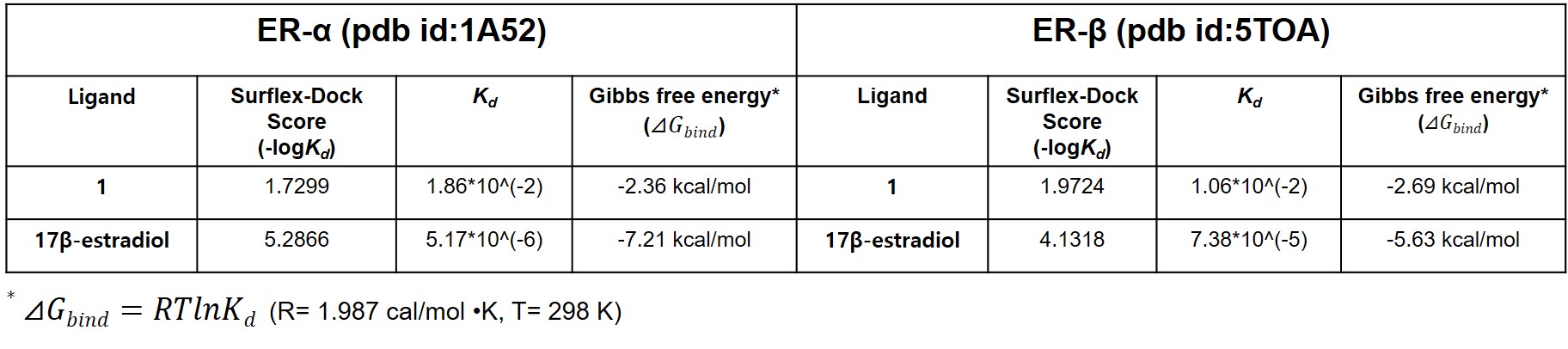
**

**Figure S8.** Calculated Gibbs free binding energy for **1**:ER versus 17β-estradiol:ER complex.


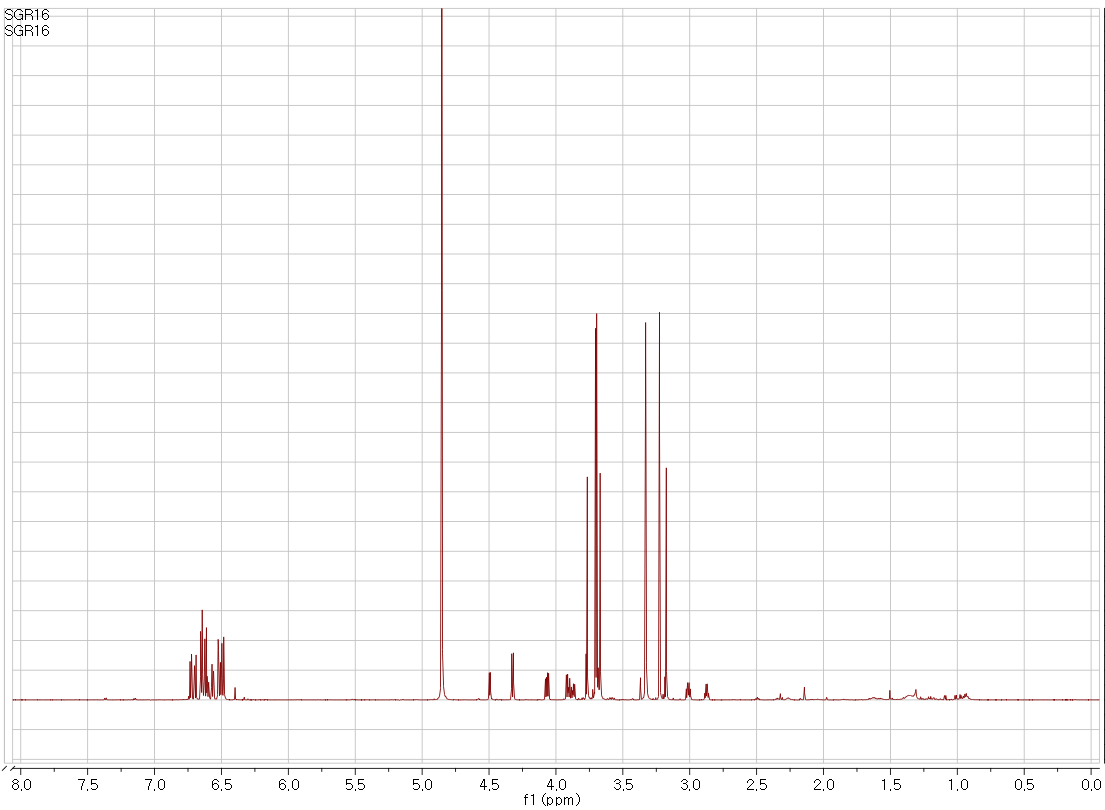


**Figure S9.** The ^1^H NMR spectrum of **2** (CD_3_OD, 800 MHz).


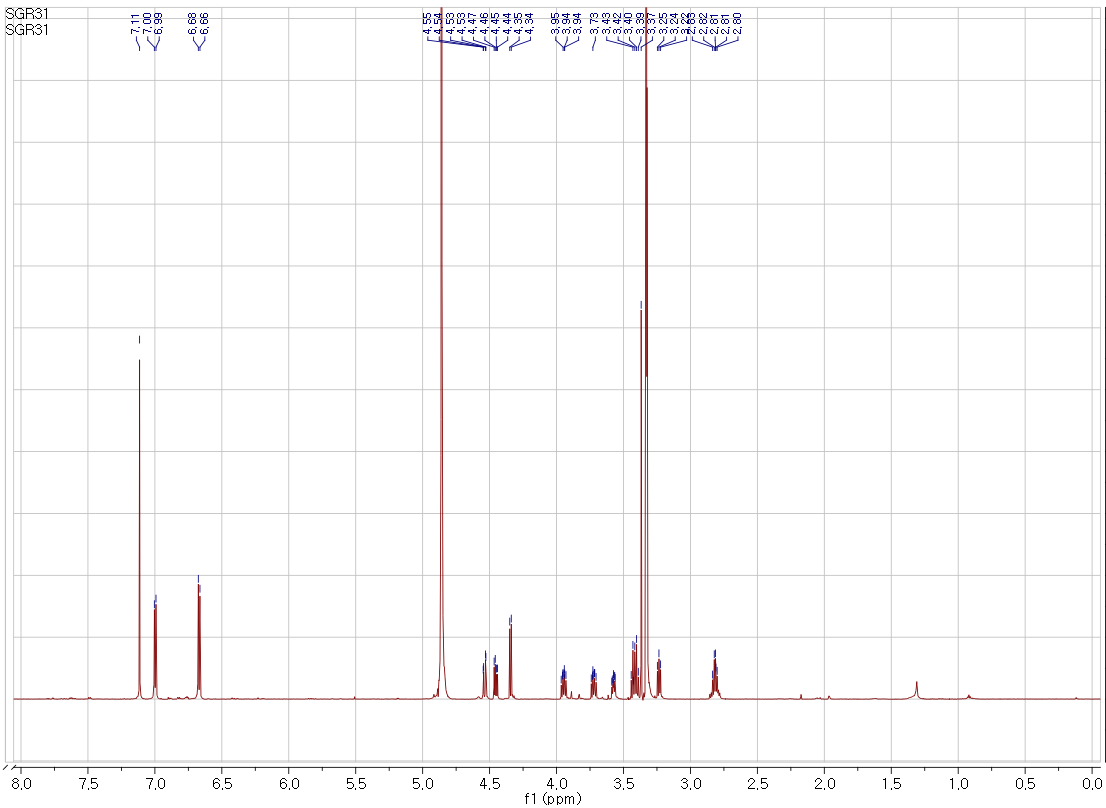


**Figure S10.** The ^1^H NMR spectrum of **3** (CD_3_OD, 800 MHz).


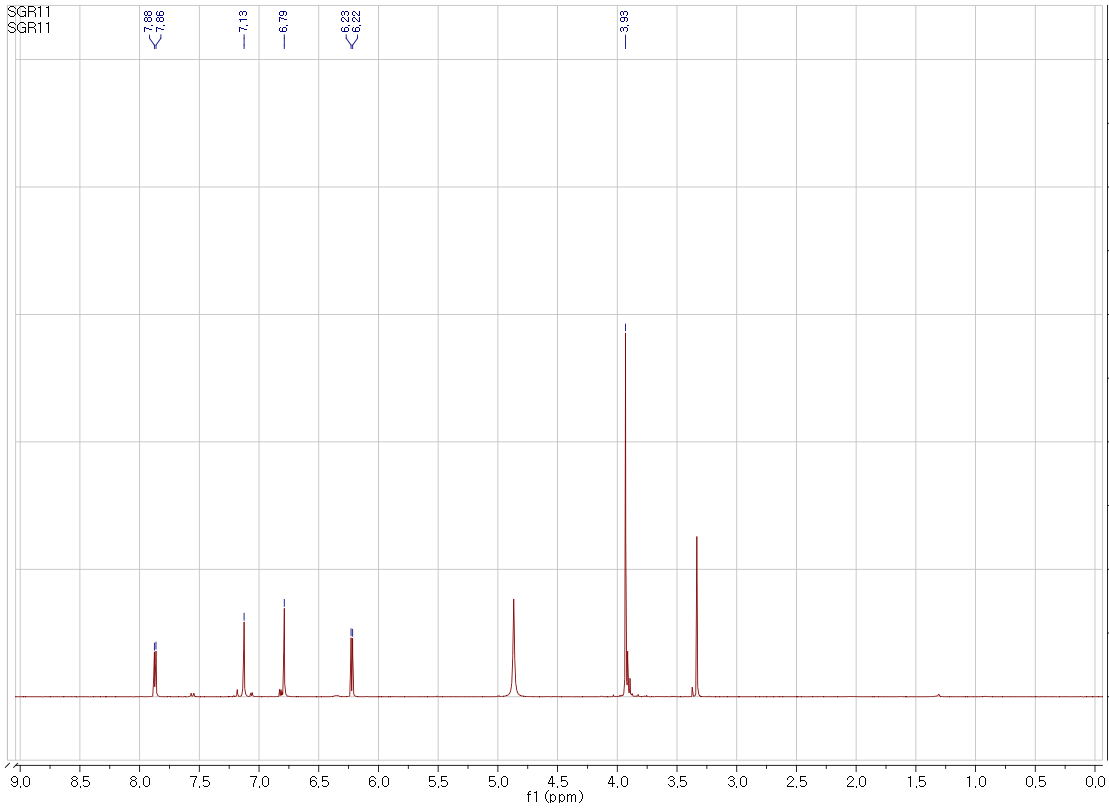


**Figure S11.** The ^1^H NMR spectrum of **4** (CD_3_OD, 800 MHz).


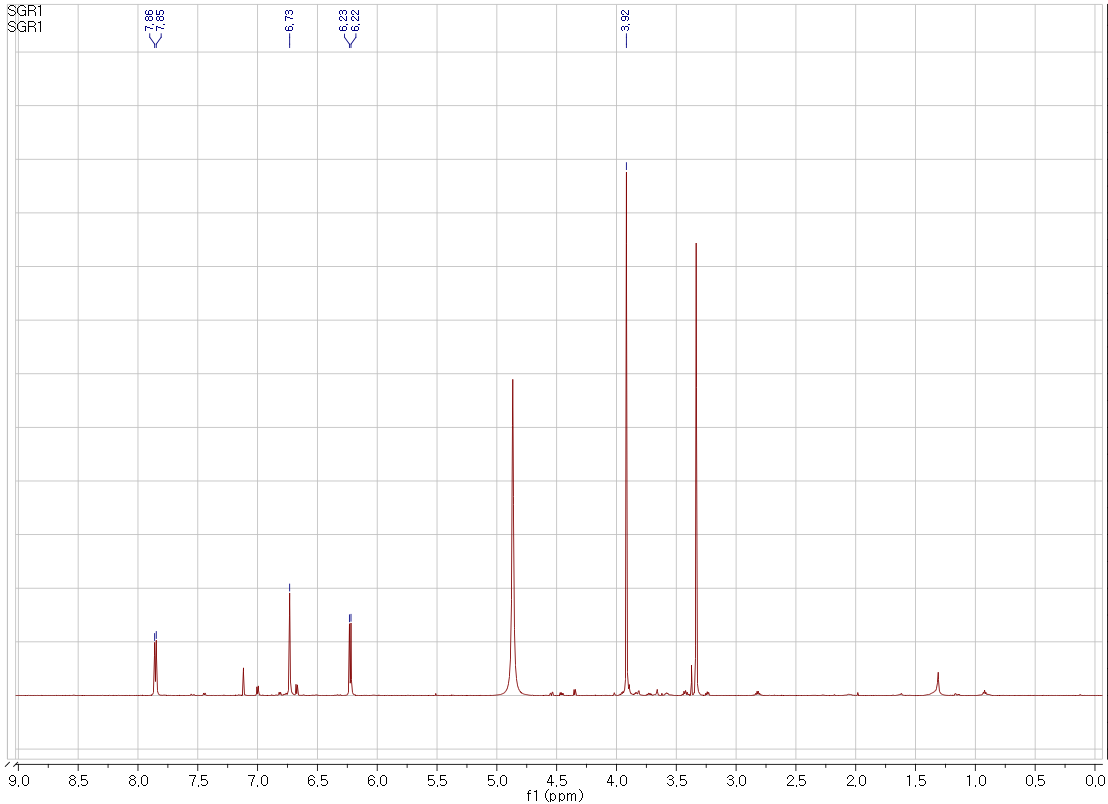


**Figure S12.** The ^1^H NMR spectrum of **5** (CD_3_OD, 800 MHz).


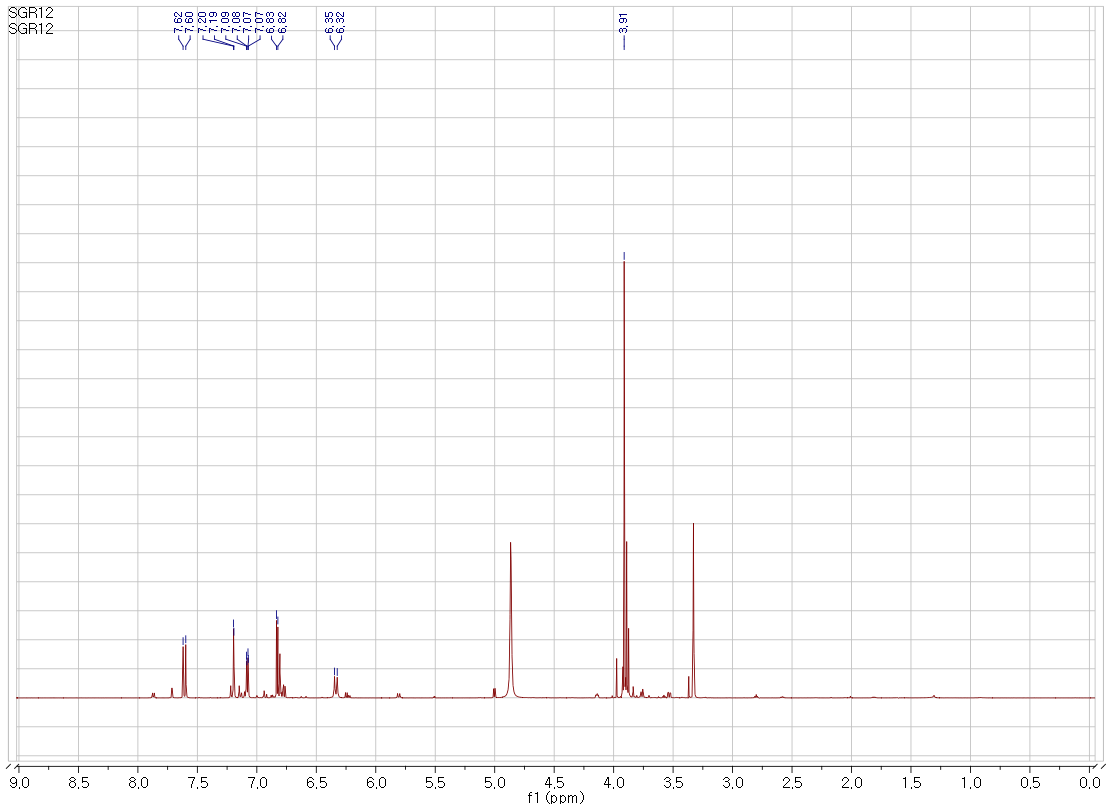


**Figure S13.** The ^1^H NMR spectrum of **6** (CD_3_OD, 800 MHz).


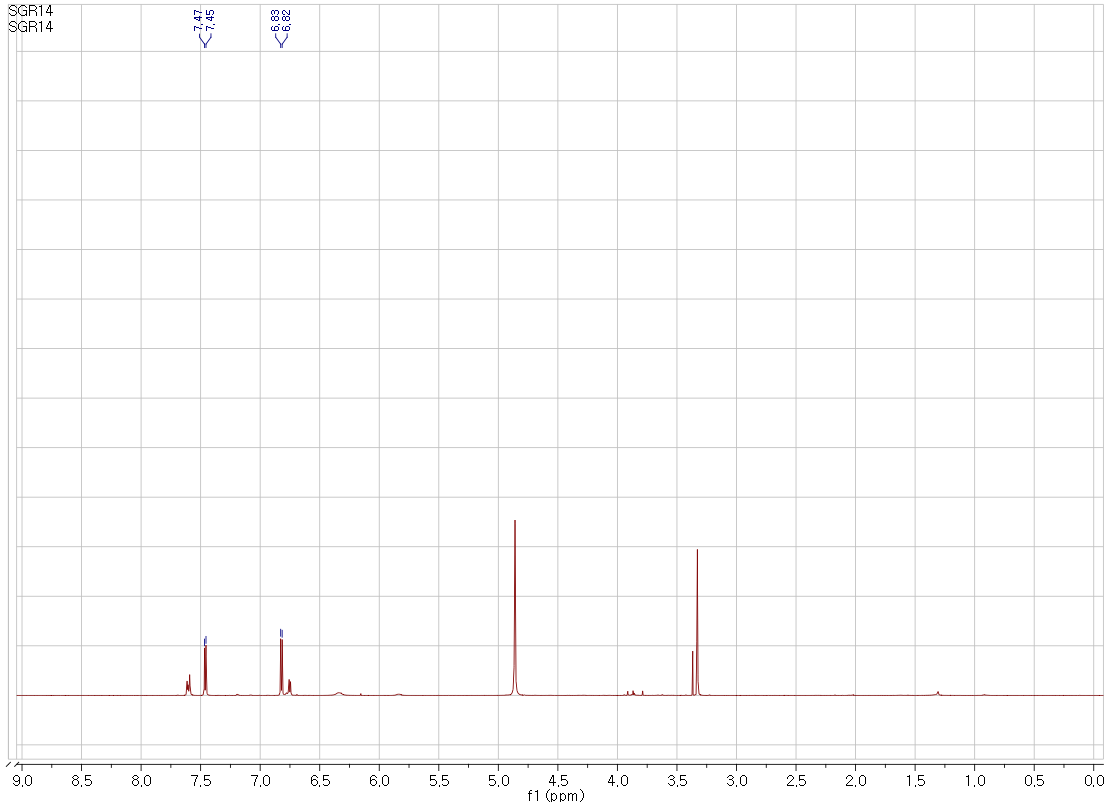


**Figure S14.** The ^1^H NMR spectrum of **7** (CD_3_OD, 800 MHz).


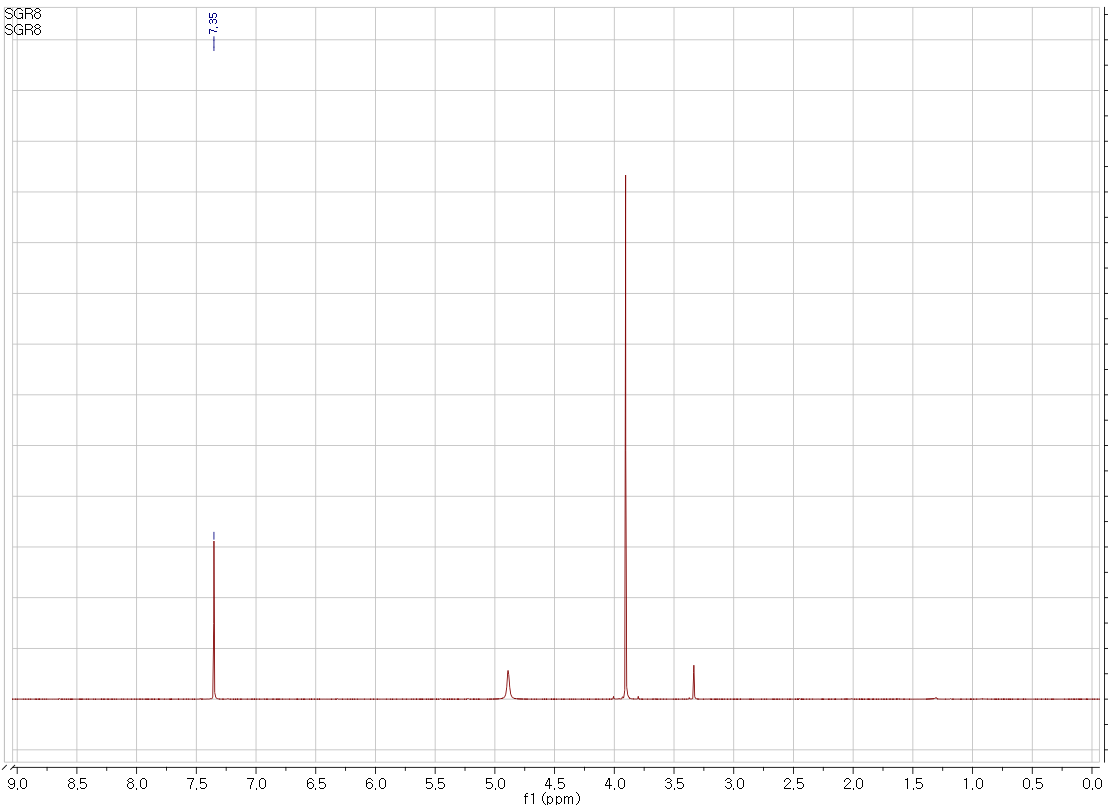


**Figure S15.** The ^1^H NMR spectrum of **8** (CD_3_OD, 800 MHz).


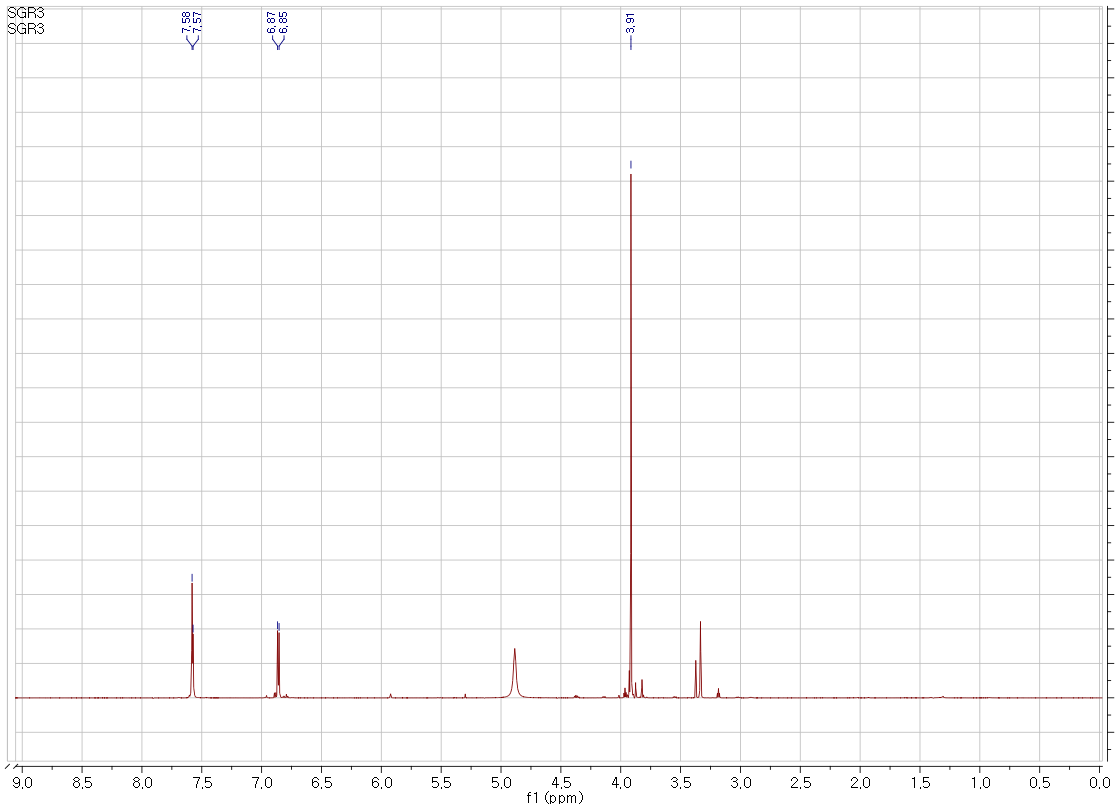


**Figure S16.** The ^1^H NMR spectrum of **9** (CD_3_OD, 800 MHz).


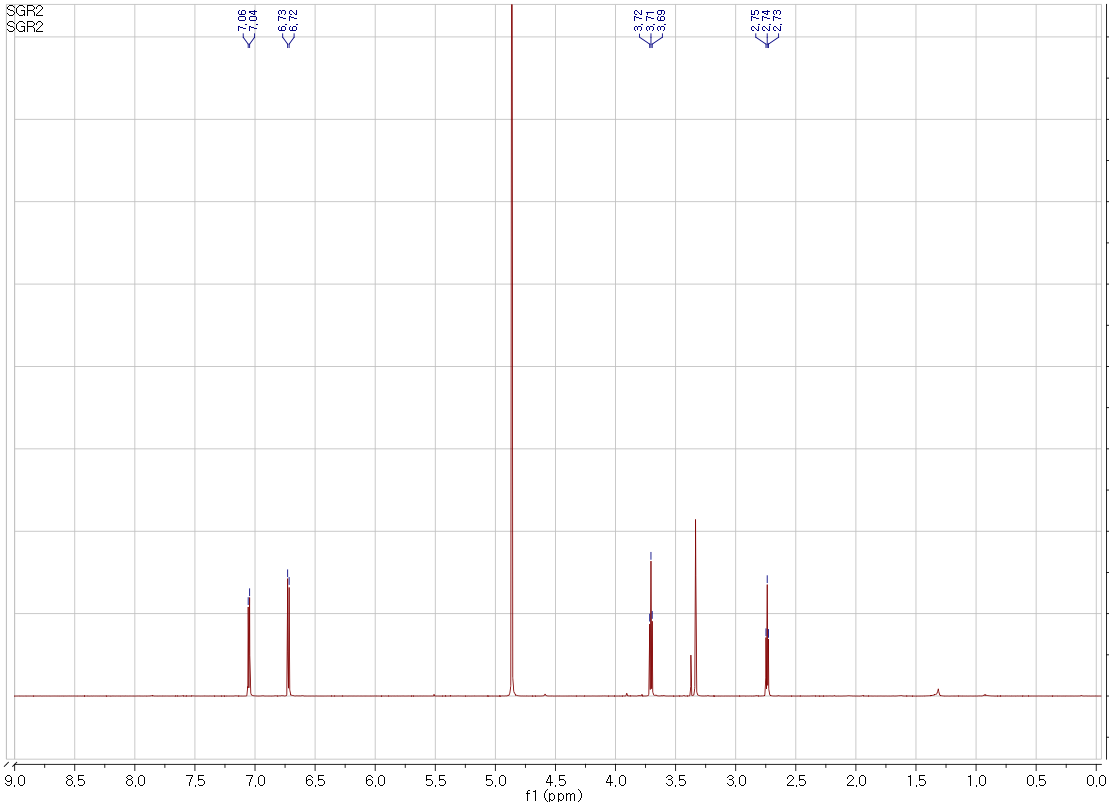


**Figure S17.** The ^1^H NMR spectrum of **10** (CD_3_OD, 800 MHz).
